# Presentation, management and outcomes of iliopsoas abscess at a University Teaching Hospital in Nepal

**DOI:** 10.1515/iss-2022-0013

**Published:** 2023-03-27

**Authors:** Jayant Kumar Sah, Shankar Adhikari, Ganesh Sah, Bikal Ghimire, Yogendra Prasad Singh

**Affiliations:** General Surgery, Maharajgunj Medical Campus, Kathmandu, Nepal

**Keywords:** drainage, iliopsoas abscess, outcome, ultrasonography

## Abstract

**Objectives:**

Iliopsoas abscess (IPA) is an uncommon clinical disease and is often missed to diagnose due to vague clinical presentation. Early treatment with drainage and appropriate antibiotic therapy is necessary before sepsis sets in and become lethal. We conducted this study to evaluate clinical features, etiology, management strategies, and outcomes in patients with IPA from a University Teaching Hospital in Nepal.

**Methods:**

A retrospective analysis of 32 consecutive IPA cases managed at Tribhuvan University Teaching Hospital, Nepal for the period of January 2019 to February 2022 was carried out.

**Results:**

The mean age was 42.5 ± 19.1 years (range, 19–75 years) and the male: female ratio was 2.2:1. Two-thirds or more patients presented with fever, limp, fixed flexion deformity and/or low back pain. Ultrasonography (US) was diagnostic in 27 (84.4%) patients. Eighteen (56.3%) patients had primary IPAs, and 14 (43.7%) had secondary IPAs. Thirty (93.7%) patients were managed with US guided percutaneous drainage (PCD) and 2 (6.2%) patients underwent open surgical drainage. Drainage procedures were combined with antibiotics in all patients. Pus culture showed *Staphylococcus aureus* growing in the majority of cases (10 of 23, 43.5%). The hospital stay was longer in patients treated via surgical drainage compared to those who received PCD: 13 days (range 12–14 days) vs. 6.6 days (range 4–13 days), respectively. Recurrence of abscess was seen in 4 (12.5%) cases and all were successfully managed via a second PCD. There was no mortality.

**Conclusions:**

Varying clinical presentation of iliopsoas abscess demand a high index of suspicion for early diagnosis. Initial imaging modality in suspected case of IPA is US. US-guided PCD along with the appropriate antibiotics is a successful frontline treatment of IPAs with shorter hospital stay.

## Introduction

Iliopsoas abscess (IPA) is a collection of purulent material in the iliopsoas compartment, which was first described by Mynter in 1881 as “psoitis” [1]. It is a rare clinical entity that is often missed by physicians due to its insidious onset and nonspecific clinical presentation [2]. IPA has been classified into primary and secondary according to its origin of infectious focus. A primary IPA reflects hematogenous spread from an occult source of infection, whereas a secondary IPA reflects contiguous spread of infection from an adjacent organ [3]. It commonly used to occur by the spread of spinal tuberculosis. However, majority of psoas abscesses have a bacterial origin since the incidence of tuberculosis is decreasing after the discovery of modern anti-tuberculosis treatment [4]. The classical triad of pain, fever, and limp, for clinical diagnosis of IPA rarely seen [5]. IPA is commonly diagnosed with the help of ultrasonography (US), computed tomography (CT), and magnetic resonance imaging (MRI) in a patient with nonspecific symptoms [6].

The management of the disease consists of appropriate antibiotics, combined with drainage of the abscess through a percutaneous or an open technique [7]. Image guided percutaneous drainage of IPA is preferable to open surgical drainage, being less invasive and associated with a shorter hospital stay [8].

The aim of our study was to review our experience with IPAs at our institution to describe this disease and assess the efficacy of currently available management strategies.

## Materials and methods

The medical records of all consecutive patients diagnosed as having IPA admitted to Tribhuvan University Teaching Hospital (TUTH) from January 2019 to February 2022, were retrospectively reviewed. TUTH serves as a referral center for tertiary specialist care in the country. Patients’ characteristics, etiologies, laboratory findings, microbiological and radiological data (US and CT), treatments, and outcomes were evaluated. The diagnosis of IPA was based on the combination of clinical features (fever, back pain, limp, flexion deformity at the hip joint), laboratory tests, and imaging (US or CT) findings.

IPA was drained using either surgical extraperitoneal approach or via image guided percutaneous drainage (PCD). Pigtail catheter (12–14 french) was used for US guided PCD depending on consistency of pus. Open surgical drainage technique was considered in case of multiloculated IPA due to unavailability of CT guided drainage facility. Intravenous broad-spectrum antibiotic was started immediately in all cases empirically; the antibiotics were later changed according to culture and sensitivity reports. Antibiotics were continued for 2–3 weeks and those who discharged earlier they were advised to continue on oral antibiotics thereafter. If drainage tube was blocked, then it was flushed with normal saline taking aseptic precaution. Drain which was kept during open and PCD procedure was removed once no further fluid came out and the abscess had completely disappeared on follow-up US. The patient who discharged with drainage tube follow up was done weekly till drainage tube removed then at one months, after this when patients became symptomatic. During follow up history, physical examination, haematological investigation, and US were done. Patients whose reports came as tuberculous abscess results received anti-tuberculous therapy and followed-up done by Orthopaedic team.

All data were analysed by SPSS for windows version 23 and simple descriptive methods were used. Quantitative data was described in mean and standard deviation considering the normality of data. The median and interquartile range was calculated in the case of skewed distribution.

## Results

A total of 32 patients were included in the study, of whom 22 (68.8%) were male and 10 (31.2%) were female, with the male to female ratio of 2.2:1 (Table 1). Mean age of presentation was 42.5 ± 19.1 years (range, 19–75 years); a majority were in the age group of 21–30 years (9 of 32, 28.1%). Eight (25%) patients had diabetes mellitus. The mean duration to diagnosis from onset of symptoms was 21 ± 15.1 days and ranged from 5 to 60 days. Fever, limp, fixed flexion deformity and back pain were the most common presenting features observed in 25 (78.1%), 24 (75), 23 (72.9) and 22 (68.7%) patients respectively. The classical triad of IPA (pain, fever, limp) was detected only in 13 patients (40.6%). Four patients were receiving anti-tubercular treatment (ATT) for spinal tuberculosis and one patients for ileocecal tuberculosis when they developed IPA. Among blood parameters, leukocytosis was seen in 24 (75%) cases with 11 (34.4%) patients demonstrating a shift to the left phenomenon. Thirty one (96.8%) patients had raised ESR values suggesting an underlying inflammatory process.

**Table 1:** Presentation of the iliopsoas abscess patients.

Characteristic	No. of patients, %
Age, years	

– Mean – Range	42.5 ± 19.1
	19–75

Gender	

– Male – Female	22 (68.8)
	10 (31.2)

Duration of symptoms, days	

– Mean – Range	– 21 ± 15.1 – 5–60

Presenting symptoms	

– Fever – Limp – Fixed flexion deformity – Low back pain – Abdominal pain – Anorexia – Loss of weight – Altered bowel habit	– 25 (78.1) – 24 (75) – 23 (72.9) – 22 (68.7) – 21 (65.6) – 9 (28.1) – 6 (18.7) – 7 (21.9)

Laboratory findings	

– Anemia (hemoglobin < 10 g/dl) – Leukocytosis (>11,000/mm^3^) – Neutrophils (>80%) – ESR > 30 mm in first hour – Hypoalbuminemia (<3.5 g/dl)	– 12 (37.5) – 24 (75) – 11 (34.4) – 31 (96.8) – 23 (71.7)

TB, Tuberculosis; ESR, Erythrocyte sedimentation rate.

**Figure 1:**
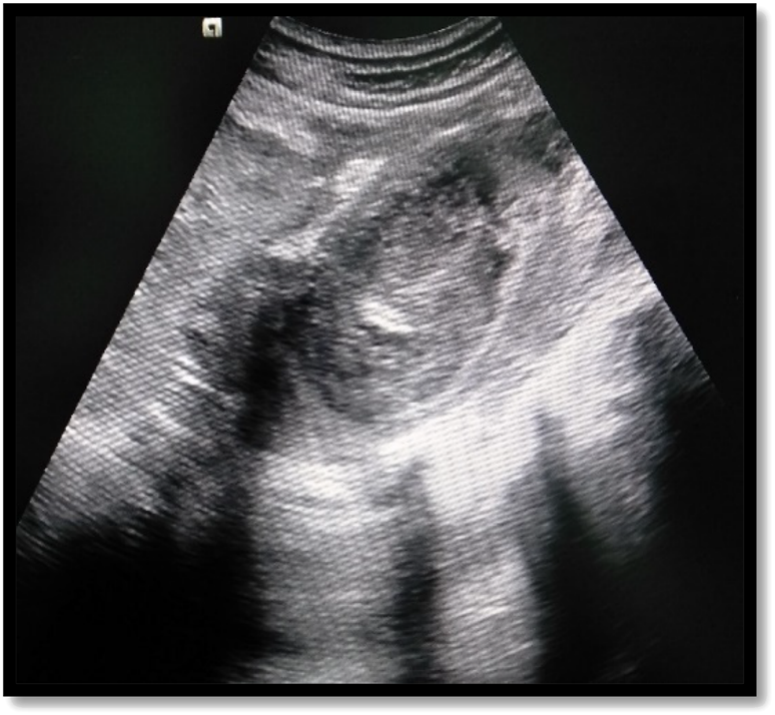
Ultrasonographic image shows heteroechoic collection in the psoas muscle.

**Figure 2:**
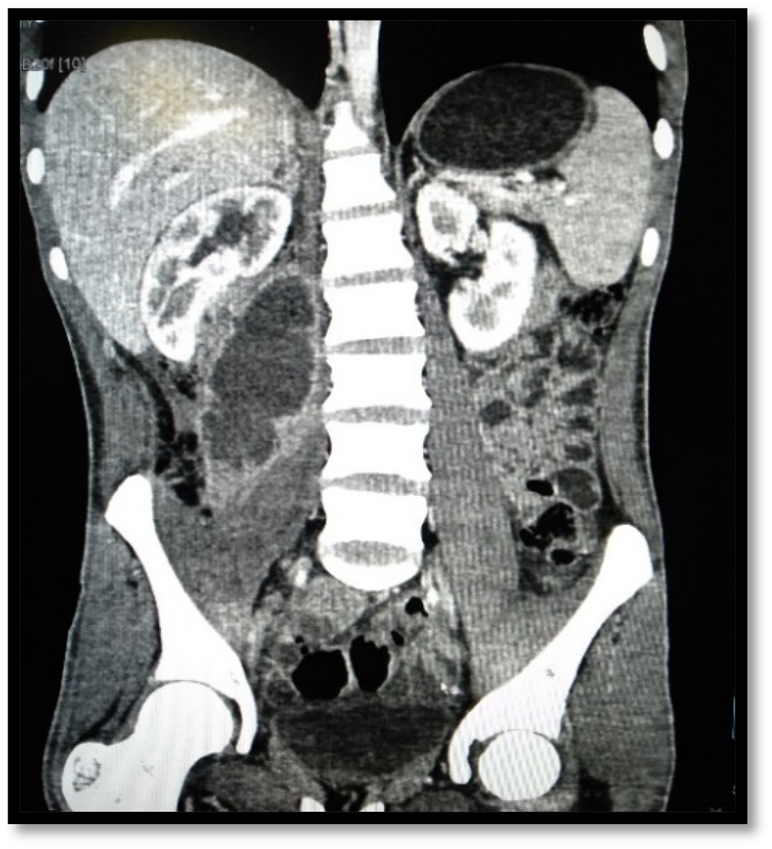
Coronal CT scan images of the abdomen and pelvis shows right sided iliopsoas abscess.

US was done in all cases of IPA, but was diagnostic in 27 (84.4%) patients (Figure 1). CT was done in 28 (87.5%) patients and MRI in 3 (9.4%) for further characterisation of abscess and underlying cause (Figure 2). Eighteen (56.3%) patients had primary IPAs without causal origin, and 14 (43.7%) had secondary IPAs (Table 2). The most common cause of secondary IPA was spinal tuberculosis 7 (21.9%). Right-sided unilateral involvement was the most common presentation in 16 (50%) cases with 12 (37.5%) cases involving left side and 4 (12.5%) cases having bilateral iliopsoas abscess. Multiple IPAs were found in 5 (15.6%). The estimated mean Volume of abscess on US was 114.1 ± 92.9 mL (range, 30–410 mL) (Table 2).

**Table 2:** Characteristics of iliopsoas abscesses.

Characteristic	No. of patients, %
Primary IPA	18 (56.3)

Secondary IPA	14 (43.7)

– Spinal tuberculosis – Urinary tract infections – Sigmoid colon diverticulitis – Ileocaecal tuberculosis	– 7 (21.9) – 4 (12.5) – 2 (6.2) – 1 (3.1)

Side	

– Unilateral – Bilateral	– 28 (87.5) – 4 (12.5)

No. of iliopsoas abscesses	

– Single – Multiple	– 27 (84.3) 5 (15.6)

Volume of abscess on US, ml	

– Mean – Range	– 114.1 ± 92.9 – 30–410

IPA, iliopsoas abscess; US, ultrasonography.

**Table 3:** Treatments and outcomes of iliopsoas abscesses.

Variable	No. of patients, %
PCD and antibiotics	30 (93.7)

– Hospital stay, (mean, days) – Recurrence	– 6.6 ± 2.5 – 4 (12.5)

Surgical drainage and antibiotics	2 (6.2)

– Hospital stay, (mean, days)	– 13 ± 1.4
Mortality	0

PCD, percutaneous drainage.

Out of 32 IPA patients, 30 patients (93.7%) received US guided PCD and only 2 (6.2%) patients underwent open surgical drainage directly (Table 3). There was no adverse events related to procedure. Cultures of the pus was positive in 23 (71.9%) IPA patients and all of these were monomicrobial infection. *Staphylococcus aureus* was the most common organism isolated from abscesses, (10 of 23, 43.5%), followed by *Esherichia coli* (8 of 23, 34.8%). Most common organism isolated in primary IPA was *S. aureus* (10 of 18, 55.5%). Table 4 shows the microbial organisms isolated and their pattern of sensitivity to the most commonly used drugs in our Hospital. *Staphylococcus* aureus was mostly sensitive to Cloxacillin and Gentamicin, and *E. coli* was mostly sensitive to Aminoglycosides (Gentamicin, Amikacin).

**Table 4:** Microorganisms isolated from iliopsoas abscess.

Culture and sensitivity/Acid fast tuberculous bacillus	No. of patients, %
Subjects with pathogens	23 (71.9)

– Monomicrobial – Polymicrobial	– 23 (71.9) – 00

*Staphylococcus aureus*	10

– Cloxacillin – Gentamicin – Piperaciilin + tazobactum – Cephalexim	– 10 – 09 – 09 – 07

*Esherichia coli*	08

– Gentamicin – Amikacin – Meropenem – Piperacillin + tazobactum	– 08 – 07 – 06 – 02

*Enterococcus faecalis*	03

– Amoxicillin – Piperacillin + tazobactum – Gentamicin – Levofloxacin	– 03 – 03 – 02 – 01

*Klebsiella pneumonia*	

– Amikacin, ceftazimide, Piperacillin + Tazobactum, Polymixin	01

*Pseudomonas aeruginosa*	

– Gentamicin, amikacin, ceftazimide, meropenem, colistin	01
*Mycobacterium tuberculosis*	08

The mean duration of hospital stay was 7.1 ± 2.9 days (range 4–14 days). The hospital stay period was longer in patients treated via surgical drainage compared to those who received PCD: 13 ± 1.4 days (range 12–14 days) vs. 6.6 ± 2.5 days (range 4–13 days), respectively. The outpatient follow-up period was 1–3 months. Recurrence of abscess was seen in 4 (12.5%) cases. All these recurrences were seen in patients treated via PCD. Of four recurrences, three cases were of primary IPA and one case of secondary IPA (spinal tuberculosis). Recurrence of abscess was managed successfully with US guided second pigtail insertion. There was no mortality due to IPA.

## Discussion

Worldwide incidence was reported as 12 cases per year in 1992 [9]. But these days increase number of cases are being reported in the literature worldwide. Yadav et al. [10], from Nepal in 2007, Kim et al. [11], from Korea in 2013, Rodrigues et al. [12], from India in 2017, Benkhadoura et al. [2], from Libya in 2019 described a series of 36 patients, 116 patients, 43 patients, 26 patients respectively. We reviewed 32 IPA patients, who were managed at tertiary care center of Nepal. Some studies previously reported that IPA is more common in the young than the elderly patients, and in males than in females [2, 4, 9, 13]. In our study also, majority of patients were young with the mean age of 42.5 years and male to female ratio of 2.2:1.

Ricci et al., reviewed 367 cases of IPA from the world literature and found that 70% (200/286 cases) were primary IPAs [14]. Asai et al., reported that 33.3% cases were primary IPAs and 66.7% were secondary IPAs [15]. Benkhadoura et al., found that 57.7% cases were primary IPAs and 42.3% were secondary IPAs [2]. In the present study, 56.3% cases were primary IPAs and 43.7% were secondary IPAs.

Medical conditions that trigger immunosuppression (diabetes mellitus, HIV infection, steroid therapy, chemotherapy) are risk factors for the development of a primary IPA [4, 7]. None of the patients exhibited such risk factors, in a study done by Benkhadoura et al., whereas Asai et al., found 33.3% (11 of 33) patients found diabetes mellitus as underlying cause [2, 15]. We found 25% of patients had diabetes mellitus.

The most common causative organism of primary IPA was *S. aureus* (10 of 18, 55.5%), comparable to various other studies [2, 7, 14], [15], [16]. Crohn’s disease is the commonest cause of secondary IPA [5, 14]. In contrast to this, Wong et al., found that spondylitis (spondylodiscitis with disc involvement) was found to be the most common etiology of secondary psoas abscess, and no cases were deemed related to Crohn’s disease [7]. According to Benkhadoura et al., most (9/11; 81.8%) secondary IPAs were due to *Mycobacterium tuberculosis* [2]. In our study, 57.1% (8/14) secondary IPAs cases showed *M. tuberculosis*.

IPAs are frequently missed at initial presentation due to nonspecific clinical presentation. Most patients in a series by Wong et al. presented with musculoskeletal (back, hip, or flank) pain, without fever; none had the classical triad of fever, back pain, and limp [7]. The classical triad of fever, limp, and back pain is present in <30% of patients [17]. In this study, the classical triad of back pain, limp, and fever was present in 40.6%; fever (78.1%), limp (75%), back pain (68.7%) were the common symptoms, and was usually nonspecific, consistent with previous studies [2, 5, 12, 15].

Computed tomography should be done for definitive diagnosis of IPA and is considered the ‘‘gold standard’’ [13]. Wong et al., reported that, in 95% (40/42) of all cases, IPAs were diagnosed via CT scan [7]. Tabrizian et al., 89% (54 of 61) of patients were diagnosed by CT scan, consistent with our findings [5]. Asai et al. [15], 81.8% patients and Tabrizian et al. [5], 87% patients had unilateral IPA. We found, 87.5% patients had unilateral involvement.

Traditionally, surgical drainage was the treatment of choice [18]. With the advent of percutaneous drainage, first described in 1984 by Mueller et al., PCD is usually considered a first-line treatment option with success rates of around 70–90% [8, 19]. In this study, US-guided PCD cured 86.7% (26 of 30) patients and the recurrence, re-treated successfully via US-guided PCD. US-guided PCD is safe, cost-effective, widely available, minimally invasive, without radiation hazard, and real time monitoring of the entire procedure. Many interventional radiologist prefer CT guided drainage of IPA because CT demonstrates the entire abscess, which is not always possible on US due to interference of overlying bowel gas. In addition, CT allows drainage of multilocular/multiseptated abscess and better visualization of possible associated pathologies in adjacent structures [8, 20]. However, it depends on availability of CT guided drainage procedure as in our study 2 (6.2%) patients with multilocular IPA underwent open surgical drainage due to its unavailability.

Tabrizian et al., Cantasdemir et al., and Benkhadoura et al., reported mean hospital stay of 25 days, 11.5 days, and s 9.5 days respectively [2, 5, 8]. We found mean hospital stay of 7.1 days with longer hospital stay in patients treated via surgical drainage compared to those who received PCD. The shorter hospital in our study may be due to early discharge of the patients with PCD tube with follow up as outpatients.

Overall recurrence rate in present study was 12.5%; comparable to studies done by Navarro-López et al., Cantasdemir et al., Benkhadoura et al., and Yacoub et al., reported recurrence rates of 15.8, 13.6, 11.5, and 9.7% respectively [2, 8, 18, 21]. Wong et al., Asai et al., and Tabrizian et al., reported mortality rates of 14, 12.1, and 5%, respectively, whereas Rodrigues et al., and Benkhadoura et al., noted no mortality [2, 5, 7, 12, 15]. In our study, there was no mortality, possibly due to most of IPA patients were young and lacked medical conditions that trigger immunosuppression.

The limitation of our study is that it is a retrospective analysis in a very small population, so its outcome is not reflective of situation of the entire community. We were unable to measure all the factors. Some of the cases classified as primary may have actually been secondary to an undetected source of infection.

## Conclusions

Iliopsoas abscess is not a rare disease and usually presents with non-specific symptoms. High index of suspicion is required to diagnose this condition early. US and CT scan are useful imaging modality for the diagnosis and management of IPA. Aminoglycoside like gentamicin and amikacin should be used as a first-line drug for empirical coverage. US-guided percutaneous drainage confers a safe, effective approach and is the main treatment along with the appropriate antibiotics.

## Supplementary Material

Supplementary Material
