# Supplementary material for: Presentation, management and outcomes of iliopsoas abscess at a University Teaching Hospital in Nepal
Source: Innov Surg Sci. 2023 Mar 27;8(1):17–22. doi: 10.1515/iss-2022-0013 (PMC10576548; doi:10.1515/iss-2022-0013)
Supplement: Supplementary file 1 — Supplementary Material [file j_iss-2022-0013_suppl_001.pdf]

Reviewer Assessment

J. Sah et al.: Presentation, management and outcomes of iliopsoas abscess at a University teaching hospital in Nepal

Reviewers' Comments to Original Submission

Reviewer 1: Mothes, Henning

Date received: 28-May-2022
Reviewer recommendation: **Return to author for minor modifications**
Reviewer overall scoring: **Medium**

Comments to author:
The spelling (especially table 4) and some minor mistakes need corrections (2/48 no Tb abbreviation in the table...) Please, avoid using phrases like developing and developed countries (18/24-26).

I disagree with two conclusions or therapy:
Why do you conclude, that "CT should be done" (19/31). If you may clearly see the abscess via USS (otherwise you may not be able to drain it). It would be an important message for other (LMIC) hospitals, that they should perform USS to detect/exclude ICA (if clinically not clear), as most hospitals in the world do not have CT scan.
Why do you treat pt´s with antibiotics for 2-3 weeks. It does not make sense. If well started with Gentamicin (which you probabaly do) and then change to an oral (for Staph aureas only).
But my main question is: Mycobacterium tuberculosis is not among the detected bacteria in your table. But in the Discussion you mention, that it was detected in 8/14 pt´s with secondary IPA, refering to ref [2], but ref [2] is Benkhadoura et al. which had 9/11 tb cases in his study. There must be a mixup of numbers or references. Please, correct.
Your text:
"According to Benkhadoura et al, most (9/11; 81.8%) secondary IPAs were due to Mycobacterium tuberculosis. In our study, 57.1% (8/14) secondary IPAs cases showed Mycobacterium tuberculosis[2]."

Reviewer 2: anonymous

Date received: 31-Jul-2022
Reviewer recommendation: **Return to author for minor modifications**
Reviewer overall scoring: **Medium**

Comments to author:
General:
See the comments in the pdf attached and make the respective corrections.
Please replace the axial CT image (see comment in the attached file).

- Material and methods
1 Any description of the type, diameter and number (per case) of drainage catheters is missing, please add.

2 Information is missing on the post-procedure protocol (rinsing the drainage? if yes: how? partially retracting the drainage before removal? imaging before removal?).
3 The authors do not mention why CT-guided drainage was not taken into consideration, especially in the cases with multilocular abscesses which have been primarily treated by open surgery.
4 There is no information given on the type of outpatient follow-up (physical exam, interview, imaging....).

- Results
5 The authors mention 7 cases of tuberculosis, but none of these cases is mentioned in table 4; please add.
6 There is no information given on the follow-up rate.

- Discussion
7 The authors do not discuss that CT-guided drainage is a widely used option for treatment of iliopsoas abscesses having the significant advantage in cases with septated and/or multilocular abscesses. Please add in the discussion and provide some references.

Authors' Response to Reviewer Comments

Date received: 06-Aug-2022

We have corrected the manuscript entitled "Presentation, management and outcomes of iliopsoas abscess at a University teaching hospital in Nepal" (Original article) according to valuable suggestions given by the reviewers. Revised manuscript have been uploaded.

Reviewers' Comments to Revised Submission

Reviewer 2: anonymous

Date received: 24-Sep-2022
Reviewer recommendation: **Return to author for minor modifications**
Reviewer overall scoring: **Medium**

Comments to author:

I refer to my first review. In the revised version, the authors did not consider my comments on CT-guided drainage:

- Material and methods
The authors do not mention why CT-guided drainage was not taken into consideration, especially in the cases with multilocular abscesses which have been primarily treated by open surgery.

- Discussion
The authors do not discuss that CT-guided drainage is a widely used option for treatment of iliopsoas abscesses having the significant advantage in cases with septated and/or multilocular abscesses. Please add in the discussion and provide some references.

Authors' Response to Reviewer Comments on Revised Submission

Date received: 18-Nov-2022

We have corrected the manuscript entitled "Presentation, management and outcomes of iliopsoas abscess at a University teaching hospital in Nepal" (Original article) according to valuable suggestions given by the reviewers. Revised manuscript have been uploaded.

Reviewers' Comments to Revised Submission

Reviewer 2: anonymous

Date received: 20-Nov-2022
Reviewer recommendation: **Accept in present form**
Reviewer overall scoring: **Medium**

Comments to author:

The comments on CT-guided drainage now have been considered by the authors.
